# High cell density cultivation of *Escherichia coli *K4 in a microfiltration bioreactor: a step towards improvement of chondroitin precursor production

**DOI:** 10.1186/1475-2859-10-10

**Published:** 2011-02-16

**Authors:** Odile Francesca Restaino, Donatella Cimini, Mario De Rosa, Angela Catapano, Mario De Rosa, Chiara Schiraldi

**Affiliations:** 1 Department of Experimental Medicine, Section of Biotechnology and Molecular Biology, Second University of Naples, Via de Crecchio 7, 80138, Naples, Italy

## Abstract

**Background:**

The bacteria *Escherichia coli *K4 produces a capsular polysaccharide (K4 CPS) whose backbone is similar to the non sulphated chondroitin chain. The chondroitin sulphate is one of the major components of the extra-cellular matrix of the vertebrate connective tissues and a high value molecule, widely employed as active principle in the treatment of osteoarthritis. It is usually obtained by extraction from animal tissues, but the risk of virus contaminations, as well as the scarceness of raw material, makes this productive process unsafe and unable to satisfy the growing market demand. In previous studies a new biotechnological process to produce chondroitin from *Escherichia coli *K4 capsular polysaccharide was investigated and a 1.4 g·L^-1 ^K4 CPS concentration was reached using fed-batch fermentation techniques. In this work, on the trail of these results, we exploited new fermentation strategies to further improve the capsular polysaccharide production.

**Results:**

The inhibitory effect of acetate on the bacterial cells growth and K4 CPS production was studied in shake flask conditions, while a new approach, that combined the optimization of the feeding profiles, the improvement of aeration conditions and the use of a microfiltration bioreactor, was investigated in three different types of fermentation processes. High polysaccharide concentrations (4.73 ± 0.2 g·L^-1^), with corresponding average yields (0.13 ± 0.006 g_K4 CPS_·g_cdw_^-1^), were obtained; the increase of K4 CPS titre, compared to batch and fed-batch results, was of 16-fold and 3.3-fold respectively, while average yield was almost 3.5 and 1.4 fold higher.

**Conclusion:**

The increase of capsular polysaccharide titre confirmed the validity of the proposed fermentation strategy and opened the way to the use of the microfiltration bioreactor for the biotechnological production of chondroitin.

## Background

Hyaluronic acid (HA), heparin (HS) and chondrotin sulphate (CS) are primary constituents of eukaryotic extra-cellular matrix of connective tissues, involved in important biological roles and in fundamental physiological processes [1]; but these glycosaminoglycans (GAGs) have also important pharmacological properties and numerous biomedical applications. GAGs are widely used as the active principle of numerous drugs [2-4] and they are nowadays considered high value molecules. They are traditionally produced by extraction and purification from animal tissue sources, such as rooster combs (HA), bovine trachea or shark fins (CS) and pork intestinal mucosa (HS), by using complex manufacturing processes that include enzymes, acidic and/or alkaline treatments and organic solvents [3,5]. The scarcity of raw materials (e.g. the shark fin cartilage) and a very high risk of viral contaminations, dangerous for human health, are the main disadvantages of this extractive method of production. All these issues may induce the regulatory officer to favour the introduction of novel biotechnological productive methods. For these reasons in the last years new approaches based on the use of microorganisms for the glycosaminoglycan production were investigated in order to meet the growing market demand, to solve the problems related to the extractive production process and to satisfy the customer's expectation to have a safe product, free from any contaminations dangerous for health. Capsulated Gram-positive and Gram-negative bacteria, whose polysaccharide layers resemble vertebrate glyco-conjugate molecules, have gained biotechnological research attention as potential GAGs producers. Various wild type or genetically modified strains of group C of *Streptococcus *genera were already employed for the biotechnological large scale production of HA by using fermentation technologies; the produced polysaccharide satisfies the market requests and it is widely employed in pharmaceutical and cosmetic products [6]. *Escherichia coli *O5:K4:H4 produces a capsular polysaccharide (K4 CPS) whose repeating disaccharide unit is constituted by glucuronic acid and N-acetylgalactosamine and that, except for a β-linked terminal furanose residue of fructose, is similar to the non sulphated chondroitin chain [7]. As we have already investigated in a previous paper [8], the capsular polysaccharide could be biotechnologically produced using *E. coli *K4 bacteria, that synthesize and release it in the culture medium during the growth. As part of the cell wall, the capsular polysaccharide could be considered a biomass related product, but its biosynthesis is strictly regulated by environmental and growth conditions [7,8]. For these reasons, the achievement of high biomass concentrations and the optimization of growth parameters are the main targets to obtain high K4 CPS yields for an efficient, economically reliable and industrially advantageous biotechnological productive process. High cell density cultivation techniques (HCDC) are commonly used in numerous manufacturing processes to reach the cost effective production of desired products, the primary goal of fermentation research [9]. The research on high cell density cultivation techniques generally includes the optimization of the feeding and aeration profiles during the fermentation process, the design of bioreactors and the study of strategies to avoid the biosynthesis of growth inhibitory by-products. For example, acetate accumulation is one of the obstacles in obtaining high product yield and productivities in cultivation of *E. coli *genus strains; this metabolic by-product is over-produced when the up-take of carbon source is greater than its conversion into biomass and CO_2_, or when the carbon flux, into the central metabolic pathway, exceeds the biosynthetic demand and the cell capacity to produce energy. In these conditions a saturation of the tricarboxylic acid cycle (TCA) and/or of the respiratory chain occurs [10]. Thus acetate is synthesized under anaerobic conditions but also under fully aerobic conditions in case of an excess of carbon sources; these two mechanisms are often referred to as mixed-acid fermentation and as overflow metabolism. Modifying the medium composition, avoiding carbon accumulation, improving the oxygen transfer capacity and controlling the feed rate are some of the bioprocess strategies used to avoid acetate accumulation; but using low feeding profiles could give also low growth rates, very low productivity and could not help in obtaining high cell density cultivations. Removing inhibiting by-products from the vessel during the fermentation processes, instead, could be a helpful way to achieve the challenge of keeping feed rates high. To reach this target various techniques have been studied and different bioreactors have been designed and used also for *E. coli *fermentation, like dialysis-fermentors or macroporous ion-exchange resin devices [11-13].

Our research group developed a microfiltration (MF) bioreactor in which two polymeric hollow fibre modules were inserted in a fermentation vessel and were able to remove low molecular weight inhibiting metabolites without removing the cells. This reactor has been already successfully employed in the growth of a recombinant *E. coli *strain, expressing a heterologous α-glucosidase from a thermophile bacteria or in the exopolysaccharides production of probiotic lactic acid strains [14,15]. In a previous paper we investigated the physiological conditions of *E. coli *K4 growth and of the capsular polysaccharide production, designing a balanced, nutrient and economic medium, suitable for large scale industrial applications. Lab-scale batch and fed-batch fermentation experiments were performed, allowing us to test a real possibility of a biotechnological production of CS from the K4 CPS and to reach a polysaccharide concentration of 1.4 g·L^-1 ^with a yield of 0.091 g_K4 CPS_·g_cdw_^-1 ^[8]. However in fed-batch experiments, after 22-24 hour feeding, the *E. coli *K4 growth and thus K4 CPS production seemed to be limited by acetate formation.

In this research, we investigated the *E. coli *K4 acetate inhibiting threshold in shake flasks conditions and we exploited the use of the microfiltration bioreactor for the production of *E. coli *K4 capsular polysaccharide, modulating the feeding and the aeration profiles in order to obtain higher polysaccharide concentration and production yield values.

## Results

### Shake flask experiments

**Figure 1:**
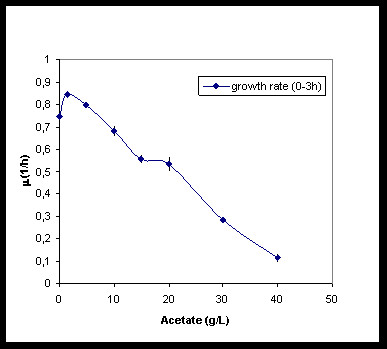
**Acetate inhibition in shake flasks**. Effect of different acetate concentration addition to the conventional medium on the specific growth rates (μ_max_) of *E. coli *K4 during exponential phase, in shake flask conditions.

**Figure 2:**
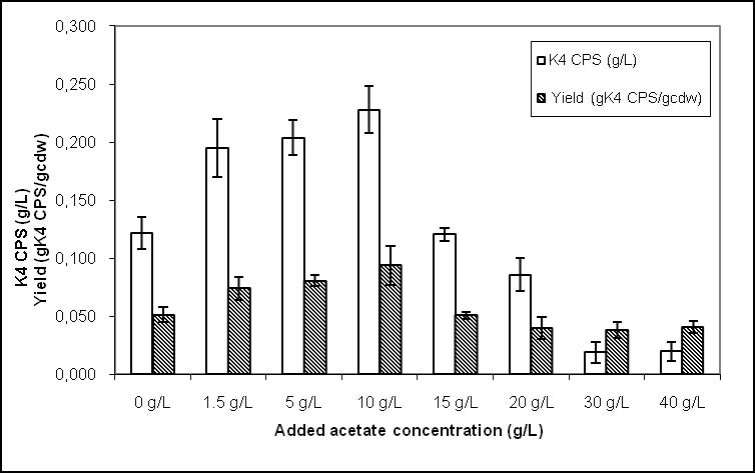
**Effect of medium supplementation with acetate on the production of K4 CPS**. K4 CPS concentration and yield on biomass, after 24 hours of growth, on conventional medium supplemented with different acetate concentrations, in shake flask conditions.

In order to investigate the effect of acetate concentration on the growth of *E. coli *K4 and its capsular polysaccharide production ability, several shaking flask experiments were performed by using eight different acetate concentrations in the medium, in the range from 0 to 40 g·L^-1^. No sensible changes in the growth medium initial pH values were observed when acetate was added (from a pH value of 7.42 ± 0.01 for 0 g·L^-1 ^to 7.35 ± 0.01 for 40 g·L^-1 ^of added acetate). Growth rates (μ_max_) calculated during the exponential phase, showed a dependency on the acetate concentrations: compared with the control (without acetate the μ_max _μ= 0.746 ± 0.055·h^-1^), the addition of 1.5 g·L^-1 ^of acetate to the medium seemed to slightly help bacterial replication increasing the μ_max _to 0.845 ± 0.017 h^-1 ^(Figure 1). A growth rate value similar to the control experiment was observed in case of addition of 5 g·L^-1 ^acetate (μ_max _μ= 0.795 ± 0.013 h^-1^), while a significant decrease in μ_max _(0.680 ± 0.022 h^-1^) was noticed when 10 g·L^-1 ^of acetate were added (Figure 1). The capsular polysaccharide biosynthesis seemed to be influenced by the acetate addition too (Figure 2). Compared to the K4 CPS concentration in the conventional medium (0.117 ± 0.01 g·L^-1^) after 24 hours of growth, the addition of 1.5 g·L^-1 ^of acetate caused an increase to 0.195 ± 0.02 g·L^-1 ^(Figure 2). Similarly to the control, in this case, the whole glycerol amount was consumed during the growth (data not shown). Cultivation on 5 g·L^-1 ^acetate added medium caused a similar K4 CPS production (0.204 ± 0.01 g·L^-1^) while on 10 g·L^-1 ^even a slightly higher titre was observed (0.228 ± 0.02 g·L^-1^). Further increasing the acetate concentration in the medium reduced μ_max_, the final biomass yield and, consequently, the K4 CPS production that, at the highest acetate concentration tested (40 g·L^-1^), decreased to 0.020 ± 0.009 g·L^-1 ^(Figure 2). In these cases the bacteria did not use the whole initial amount of glycerol (residual concentration between 3.2 and 3.9 g·L^-1^) and did not reach the same biomass maximum value obtained in the conventional medium. As already reported before [8], in all shake flask experiments, K4 CPS concentration reached the maximum after 24 hours of culture. Different experiments tested the ability of *E. coli *K4 to grow on acetate (from 1.5 to 20 g·L^-1^) in glycerol free medium. Also in this case the acetate addition did not cause any pH modification in the media. The bacteria metabolised acetate, as visible in Table 1, and a residue of acetate was observed at the end of the experiment only in case of the highest initial concentration. The higher the acetate titre was the greater the cell concentration achieved (passing from 0.92 to 1.99 g_cdw_·L^-1^), while growth rates quickly diminished from 0.84 to 0.51 h^-1^.

**Table 1:** *E. coli *K4 shake flask experiments on acetate

**Initial acetate concentration **(g·L^-1^)	**1.5**	**5**	**10**	**20**
**Final acetate concentration **(g·L^-1^)	0.031	0.38	0.98	5.25
**Biomass **(g_cdw_·L^-1^)	0.92	1.57	1.98	1.99
**μ max **(h^-1^)	0.84	0.69	0.64	0.51

*E. coli *K4 shake flask growth on 1.5, 5, 10, 20 g·L^-1 ^acetate media without glycerol: initial and final acetate concentration, max biomass and max μ values. All the standard deviations were less than 6%.

### Batch experiments

**Figure 3:**
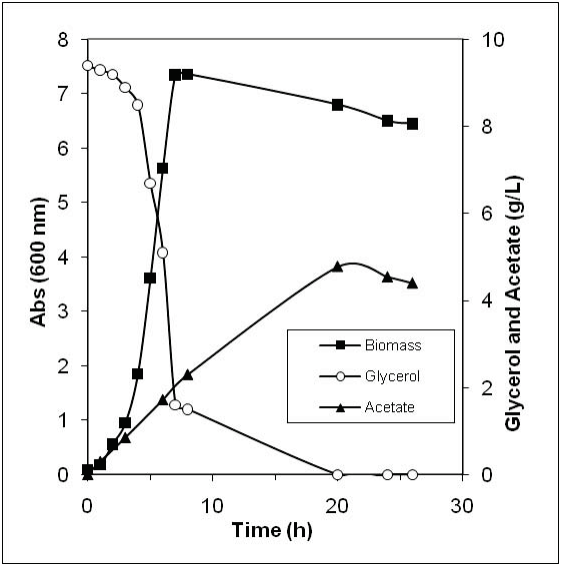
**Batch experiments**. *E. coli *K4 batch experiments: growth, glycerol consumption, acetate production and re-consumption.

Fermentation experiments were performed in batch conditions on the conventional medium to investigate K4 CPS production, as previously described [8]. Glycerol and acetate concentrations during growth are reported in Figure 3. The glycerol consumption rate ranged between 1.1 and 1.4 g·L^-1^·h^-1^, while acetate was produced at 0.30 ± 0.02 g·L^-1^·h^-1^. Successively, in late stationary phase, acetate slowly decreased with a consumption rate in the range from 0.06 to 0.07 g·L^-1^·h^-1^.

### Microfiltration experiments

**Figure 4:**
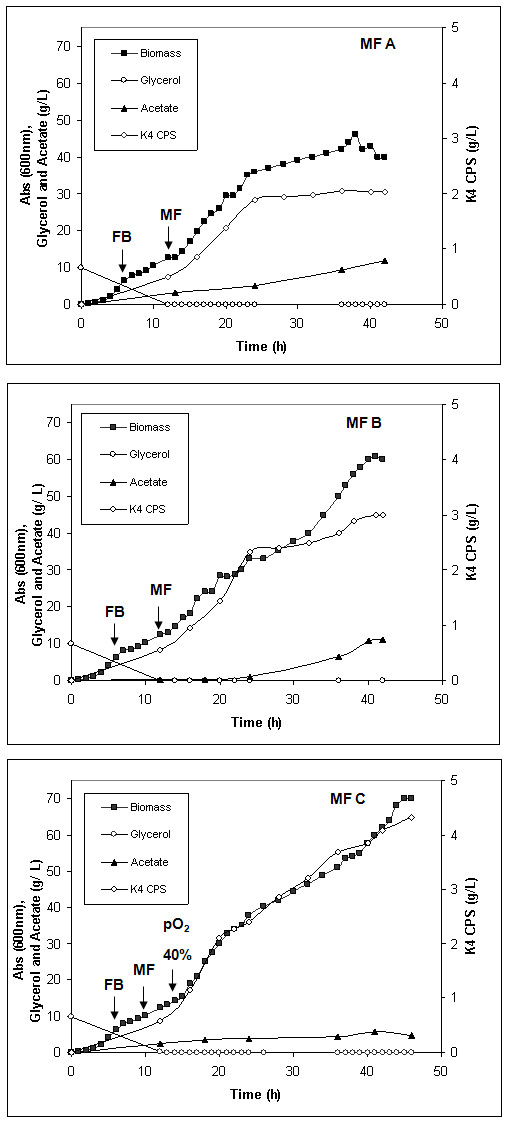
**Microfiltration experiments**. *E. coli *K4 three different MF experiments: growth, glycerol consumption, acetate and K4 CPS production. The arrows indicate the starting points of fed-batch phase (FB), microfiltration phase (MF) and of the eventual increase of the pO_2 _setting (pO_2 _40%).

**Figure 5:**
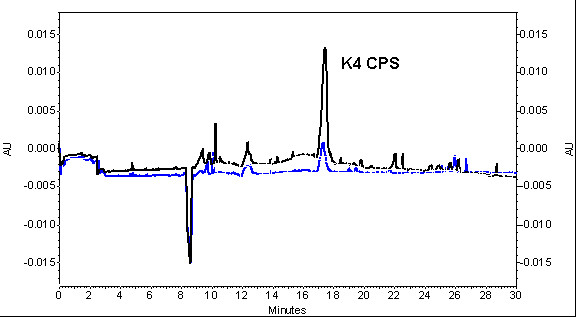
**Capillary electrophoresis analysis of K4 CPS in microfiltration supernatants and permeates**. Overlaid electropherograms of *E. coli *K4 CPS in the MF broth supernatants (black trace) and in the permeates (blue trace).

**Figure 6:**
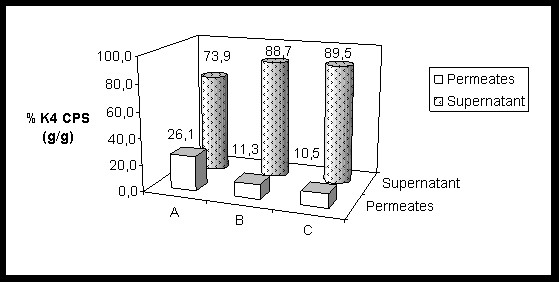
**K4 CPS presence in microfiltration supernatants and permeates**. *E. coli *K4 three different MF experiments: the ratio, in percentage, between the K4 CPS amounts (g) present in the supernatants or in the permeates on the total amount (g) of K4 CPS produced, at the end of the growth. All the standard deviations are less than 6%.

Three different types of microfiltration experiments were performed to study the influence of carbon source concentration in the medium and of a good oxygenation on bacterial growth, on capsular polysaccharide production and on acetate formation. The design of the feeding profiles and of the aeration conditions was coupled with the *in situ *product removal strategy. First of all, we investigated two different feeding profiles to find the best one for both growth and K4 CPS production. In all the experiments identical batch and fed-batch conditions were set, the modules were activated after 12 hours of growth and identical feeding profiles were used up to 14 hours (see Materials and Methods). After that, two different feeding rates, were designed and tested in the MF experiments (Table 2). In MF A profile, the feed rate of glycerol, supplied to the culture, reached a maximum value of 4.5 g·L^-1^·h^-1^. As visible in the graph (Figure 4-MF A), all the glycerol fed (total amount 231.5 g) was consumed by the bacteria without accumulation and a maximum biomass concentration of 23.92 ± 0.72 g_cdw_·L^-1^, with a corresponding yield of 0.186 ± 0.005 g_cdw_·g^-1^, was obtained. The final K4 CPS concentration in the culture supernatant was 2.03 ± 0.08 g·L^-1 ^(Table 3). The microfiltration modules were able to refresh the medium keeping an acetate concentration inside the vessel lower than 5 g·L^-1 ^up to 24 hours of culture; then the increasing amount of acetate (final concentration > 10 g·L^-1^) decelerated growth toward an initial stationary phase and lowered the K4 CPS production. In order to further enhance the capsular polysaccharide production, a higher glycerol feeding rate was used for the MF B experiment and improved aeration conditions were supplied sustaining the bacterial cell respiration (Table 2). The bacterial cells perfectly adapted their metabolism to the set profile, thus prolonging the growth phase up to 42 hours (Figure 4-MF B). The culture reached a higher maximum biomass concentration (31.7 ± 1.3 g_cdw_·L^-1^) and higher biomass yield (Y_x/s_= 0.197 ± 0.008 g_cdw_·g^-1^), compared to the MF A results. The final acetate in the vessel was about 10 g·L^-1 ^after 40 hours of growth and the bacteria produced 3.00 ± 0.12 g·L^-1 ^of K4 CPS (Table 3). In MF B almost the whole amount of glycerol fed to the culture was consumed by the bacteria (291.8 g glycerol fed versus 289.7 g glycerol consumed). Further increasing the glycerol feeding rate did not improve K4 CPS production (data not shown). Once the feeding profile was optimized, the pO_2 _setting was varied in order to study the effect of a higher oxygen concentration on growth and K4 CPS production. A third type of experiment (Figure 4- MF C) was conducted using the same optimised feeding profile of MF B (Table 2) but the dissolved oxygen (DO) lower limit was increased from 20% to 40% soon after 15 hours of growth. In this way the acetate formation was limited and its concentration always kept below 6 g·L^-1 ^inside the vessel. As a consequence of that a higher maximum biomass concentration and biomass yield on substrate were reached (36.4 ± 1.5 g_cdw_·L^-1 ^and 0.204 ± 0.008 g_cdw_·g^-1^), compared with the previous results, and a final K4 CPS concentration in the culture supernatant of 4.33 ± 0.17 g·L^-1 ^was obtained (Table 3). A further increase of oxygen in the vessel, setting the pO_2 _value at 70%, did not support the growth and the capsular polysaccharide production in the same way (the maximum biomass reached was 27.4 g_cdw_·L^-1 ^and the final total K4 CPS concentration was 2.33 g·L^-1^). A certain stress effect was noted and, already after 19 hours of growth, the viability of the bacterial cells resulted diminished of almost 40% compared with the one obtained in MF C fermentation (data not shown). During MF experiments the K4 CPS produced and released in the medium was partly found in the permeates (Figure 5). The whole amount of capsular polysaccharide in all the permeates, at the end of each experiment, was determined and the exact final total K4 CPS concentration was calculated (Table 3); thus the precise yields of K4 CPS on biomass and on substrate as well as the productivity values were re-calculated (Table 3). The percentage of the capsular polysaccharide found in the permeates varied according to the experiment; the higher the K4 CPS amount in the culture supernatant, the lower the one found in the microfiltrated volumes (Figure 6).

**Table 2:** Microfiltration experiment parameters

	MF A	MF B	MF C
**Stirring** (rpm)	250-600	250-1000	250-800
**Aeration** (L·min^-1^)	1.0-1.4	1.0-1.8	1.0-1.6
**pO_2 _setting** (% DO)	20	20	20-40
**Feeding profile MF phase** (g·L^-1^·h^-1^)			
12-24 hours	2.7→ 4.5	2.7→ 5.4	2.7→ 5.4
24-to the end	4.5→ 3.2	5.4→ 4.6	5.4→ 4.6
**Total glycerol fed **(g)	236.5	291.8	325.3
**Total glycerol consumed **(g)	231.9	289.7	320.0

*E. coli *K4 three different MF experiments: Stirring, aeration and pO_2 _setting parameters; feeding profiles in MF phase, total glycerol fed and consumed.

**Table 3:** Microfiltration experiment results

	MF A	MF B	MF C
**Time **(h)	42	42	46
**Maximal biomass concentration **(g_cdw_·L^-1^)	23.90	31.70	36.40
**μFB phase **(h^-1^)	0.21	0.21	0.21
**μMF phase **(h^-1^)	0.10	0.13	0.12
**Overall Y_X/Glycerol _**(g_cdw_·g^-1^)	0.186	0.197	0.204
**Culture supernatant K4 CPS concentration **(g_K4CPS_·L^-1^)	2.03	3.00	4.33
**Total K4 CPS produced **(g_K4CPS_·L^-1^)	2.73	3.43	4.73
**Overall Y_K4 CPS/Glycerol _**(g_K4CPS_·g^-1^)	0.021	0.022	0.027
**Y_K4 CPS/X _**(g_K4CPS_·g_cdw_^-1^)	0.067	0.108	0.130
**Productivity K4 CPS **(g_K4CPS_·L^-1^·h^-1^)	0.065	0.082	0.103
**J_MF 12-24 h _**[L·(m^2^·h)^-1^]	1.71	1.62	1.63
**J_MF 25h-end _**[L·(m^2^·h)^-1^]	0.88	1.1	0.91
**Permeate total volume **(L)	5.60	6.50	6.10
**Exchanged volumes **(L·L^-1^)	3.10	3.60	3.40

Parameters and data obtained from the *E. coli *K4 three different MF experiments. All the standard deviations were less than 5%.

## Discussion

Glycosaminoglycans are high value molecules, with a wide range of biomedical applications. In the last decade new biotechnological productive processes to obtain GAGs have been studied in order to avoid the traditional, long and unsafe extractive procedures from animal tissues. Hyaluronic acid has been produced commercially since the early 1980 s using *Streptococcus equi *subsp. *equi *and subsp. *zooepidemicus *or genetically modified strains [6]. Biotechnological chondroitin sulphate, instead, is not currently available on the market. In a previous paper we investigated the possibility to set up a biotechnological process for the production of chondroitin, starting from a structural related capsular polysaccharide (K4 CPS) produced by *E. coli *O5:K4:H4 [8]. In particular, a K4 CPS concentration of 1.4 g·L^-1 ^was reached in fed-batch fermentation but a further increase in biomass and capsular polysaccharide production was probably limited by organic acid formation. Acetate accumulation is one of the main constraints in *E. coli *fermentations; this growth inhibiting metabolite is produced in excess of substrate or in conditions of oxygen limitation. Different strategies are described in literature to limit its synthesis: genetic approaches variously modified the central metabolism of *E. coli*, altering the glucose uptake mechanism or the tricarboxylic acid pathways. The optimization of the media and the study of the oxygen consumption are tools generally used in the bioprocess approaches [10]. Glycerol is widely used as carbon source, instead of glucose, because of its low transport rate into the cell that helps in slowing the flux of carbon through the glycolysis thus reducing the acetate production [9]. Insufflating oxygen-enriched air could be a solution to avoid anaerobic conditions of growth, while the employment of pH-stat or substrate limited feeding profiles could control acetate formation but also reduce the bacterial cell growth rate. Dialysis and macro porous ion-exchange resin based fermentors have been widely employed in order to obtain high cell density cultivations of *E. coli*, limiting acetate inhibition and, at the same time, avoiding the drawbacks of low growth rate feeding methods [11-13]. Recently high cell density cultivations of *E. coli *for the production of recombinant proteins were performed in rocking-motion-type bioreactor [16].

A dialysis bioreactor has been used also for the growth of the capsulated strain *E. coli *K12 but the study focused on the biomass formation and not on the polysaccharide production [17]. The inhibiting effect of acetate on *E. coli *K4 growth rate could be a problem in the designing of a high cell density cultivation process for the production of the chondroitin-like capsular polysaccharide. Although the capsular polysaccharide constitutes part of the cell wall and could be considered a biomass related product, its biosynthesis is influenced by nutritional, environmental and cultivation conditions [7,8]. For these reasons we first investigated, in shake flask experiments, the effect of acetate, added to the conventional medium, and its influence on both bacterial cell growth and K4 CPS production. Our data showed that acetate concentrations higher than 5 g·L^-1 ^negatively influenced *E. coli *K4 growth rates, while K4 CPS production is positively affected up to 10 g·L^-1 ^acetate supplementation and progressively reduced by higher concentration addition (Figure 2). An acetate concentration higher than 5 g·L^-1 ^is commonly considered an inhibiting threshold for *E. coli *genus, because it reduces growth rates and biomass yield [18]; De May and co-worker even reported a decrease of growth and of recombinant protein production above 1 g·L^-1 ^[10]. *E. coli *K4 showed the same behaviour, although a higher tolerance for the acetate presence in the medium, up to 10 g·L^-1^, was observed. This higher adaptability could probably be related to the presence of the capsular polysaccharide that has a role of cell protective layer in various stressful conditions [19]. On the other hand, as reported for other strains of the same genus [20], also *E. coli *K4 seemed to be capable to metabolise acetate, growing in shake flask experiments on a medium containing up to 20 g·L^-1 ^acetate (Table 1). *E. coli *K4 metabolised acetate also in batch experiments: in the stationary phase, when glycerol was exhausted, the bacterial cells started to re-consume the produced acetate with a rate similar to the ones reported for other *E. coli *strains [21]. Although the cells can use acetate, prolonging the growth resulted not convenient. In batch, the maximum of K4 CPS titre is usually reached in 24 hours [8] and after that time lysis of the bacterial cells occurrs. These data were taken into consideration to set up and optimise the microfiltration experiments in which a balance was sought between keeping growth rates high to improve K4 CPS productivity and not accumulating acetate beyond the critical threshold (in our case between 5-10 g·L^-1^). Placing microfiltration modules in the bioreactor the exhaust medium could be removed from the vessel, while the cells were kept inside in a controlled environment. Three different types of experiments in microfiltration were run as described in the Material and Methods section. In MF A experiments a first feeding profile was tested and, at the end of the growth, a K4 CPS concentration 1.5-fold higher than the one previously reported for the fed-batch experiments was obtained [8]. In MF B experiments, instead, the use of a higher feeding profile, coupled with higher aeration rates and a medium exchange of 3.6 volumes, allowed to keep the acetate concentration in the vessel below 5 g·L^-1 ^for a longer time respect to MF A. In these conditions the bacteria produced 2.1-fold higher K4 CPS titre, without any glycerol accumulation. This feeding profile was considered the optimal one for our purposes and used also in MF C experiments. In this case O_2 _enrichment was provided to keep up with the strain's oxygen demand, without further pushing the airflow and the stirring rates that easily caused mechanical cell stress and foam formation. The oxygen setting up at 40%, after 15 hours of growth, together with the medium exchange, assured better metabolic conditions than in MF B experiments, thus maintaining the acetate concentration below 6 g·L^-1 ^up to the end of the experiment. As a result, this increase in oxygen availability permitted the achievement of a higher maximum biomass concentration and biomass yield on substrate as well as the highest K4 CPS titre ever obtained (4.33 ± 0.17 g·L^-1^) (Table 3). As reported before for the MF experiments of exopolysaccharide producing lactic acid strains [15], part of the K4 CPS, produced by the cells and then released in the medium during the fermentation, was eluted in the microfiltrated volumes. Although the MF capillaries have a cut-off of 0.22 μm, not all the K4 CPS produced was found in the permeates and the percentage of eluted capsular polysaccharide seemed to depend on the polysaccharide concentrations in the culture supernatant (Figure 5 and 6). Certainly cellular cake depositions, that became thicker as the cell density grew, contributed to the K4 CPS retention inside the vessel, together with a polysaccharide gel layer formation. In particular, in MF B and C the higher K4 CPS concentrations in the supernatants, respect to MF A, contributed to module surfaces fouling and reduced by half the flow-through of capsular polysaccharide in the microfiltrated volumes. Considering also the amount of K4 CPS in the permeates, the exact final total capsular polysaccharide production, yields and productivities for each type of experiment were re-calculated. In all the MF experiments higher values, than those previously reported, were obtained [8]; in particular, in the MF C, the highest K4 CPS concentration, so far obtained, was produced (4.73 ± 0.20 g·L^-1^), corresponding to an amount almost 16-fold and 3.33-fold higher than the ones obtained using batch and fed-batch techniques, respectively [8].

## Conclusions

Acetate concentration proved to affect bacterial cell growth and K4 CPS production. All the results of fermentation experiments confirmed the consistency of the microfiltration bioreactor technology as a valid tool in the biotechnological production of the chondroitin precursor, the *E. coli *K4 capsular polysaccharide. In the framework of this research we addressed the issue of the low yields reported for the microbial production of K4 CPS [22], permitting the achievement of titres similar to the ones obtained in the biotechnological production of the most noted glycosaminoglycan, that is hyaluronic acid [23].

## Materials and methods

### Materials

All the growth medium components, salts and the antifoam agent were supplied by Sigma-Aldrich (USA). Neutralised soya peptone was furnished by OXOID (United Kingdom), while ammonium hydroxide, sulphuric acid and phosphoric acid were purchased by Carlo Erba (Italy). Salts, acid or alkali components, needed to prepare buffers for capillary electrophoresis, for high pressure liquid chromatography and anion exchange chromatography analyses were by Sigma-Aldrich (USA).

### Bacterial strain

The strain *E. coli *O5:K4:H4 (U1-41,11307) was purchased from the Culture Collection of the University of Göteborg (Sweden). The strain was stored and maintained in 20% (v/v) glycerol stock solutions at T= -80°C. The growth medium used was the same described in a previous paper [8]; [Conventional medium composition: glycerol (10 g·L^-1^), soya peptone (1 g·L^-1^), KH_2_PO_4 _(2 g·L^-1^), K_2_HPO_4 _(9.7 g·L^-1^), sodium citrate dehydrate (0.5 g·L^-1^), (NH_4_)_2_SO_4 _(1 g·L^-1^), MgCl_2 _(0.1 g·L^-1^)]. This medium was used in shake-flask experiments after being sterilized in autoclave: in acetate inhibiting threshold tests acetate was added to the conventional medium, in acetate metabolising tests acetate was added to the glycerol depleted medium. Medium *in situ *sterilization was used for fermentation experiments. In all cases, K_2_HPO_4 _was added to the sterilized media as a concentrated solution (388 g·L^-1^) after sterilization by filtration with 0.22 μm membranes (Millipore, France).

### Shaking flask experiments

The acetate inhibiting threshold for *E. coli *K4 growth was investigated during shake flask experiments. Variable volumes of a 3.4 M acetate stock solution were added to the conventional medium, at the beginning of the growth, in order to obtain the desired final acetate concentration (0, 1.5, 5, 10, 15, 20, 30 and 40 g·L^-1^). Small aliquots (2 mL) of the growth medium, after acetate addition, were withdrawn to measure the initial medium pH by using a pH-meter (Cyberscan 510, Eutech Instruments, Thermo Fisher Scientific Inc.). For every single acetate concentration, three independent experiments were performed using 1 L Erlenmeyer flasks, equipped with four baffles and containing 200 mL of medium, in a rotary air shaker (model Minitron, Infors, Switzerland) with a rotation speed of 200 rpm at the optimal growth temperature of 37°C. Glycerol stock preparations of *E. coli *K4 were used to inoculate the media. Samples of broth were collected at time intervals of 1 hour, up to 24 hours of growth, to measure the absorbance at 600 nm (Spectrophotometer DU800, Beckman Coulter, USA). Biomass dry weight was measured during cultivations: variable volumes (2-5 ml) of cultures were filtered on a 0.22 μm propylene membrane, the pellet deposited on the microfilter was then washed with a volume of physiological saline solution and successively the membrane was placed in an oven at 80°C for 18 h to achieve constant dry weight. Cell dry weight values were then calculated and correlated in a plot to the absorbance measurements.

*E. coli *K4 growth rates (μ) in the different media were calculated during the exponential phase (from 0 to 3 hours) with a linear regression method as averaged values of three experiments. After 24 hours of culture, samples of supernatant were withdrawn to evaluate also the average K4 CPS concentration in the different growth conditions. A *E. coli *K4 growth on the complete conventional medium was performed every time as control.

Further shake flask experiments were performed to test the strain ability to metabolise acetate; variable volumes of the 3.4 M acetate stock solution were added, at the beginning of the growth, to the conventional medium, depleted of glycerol, in order to obtain an initial acetate concentration of 1.5, 5, 10 or 20 g·L^-1^. Growths were performed, as already described above, in triplicate; initial pH was checked, biomass formation was followed by measuring the absorbance at 600 nm up to 24 hours of culture and cell dry weight values were obtained as described above. Growth rates (μ) were calculated during the exponential phase (from 0 to 3 hours). Sample of supernatant were withdrawn to analyse the acetate concentrations in the media at initial and final time.

### Fermentors

A Biostat CT fermentor, (Braun Biotech International, Sartorius Group, Germany), *in situ *sterilizable, with a total volume of 2.5 L and a working one of 2 L was used to perform batch and microfiltration experiments. The system was equipped with pH, temperature and pO_2 _probes and four peristaltic pumps for the addition of alkali, acid, nutrients, salts or antifoam solutions. All the fermentation parameter data were registered by a Digital Control Unit (DCU) during the whole experiments, while a computer connected to the fermentor, equipped with a MFCS-win software, was used to remotely control all the fermentation parameters, included pH, pO_2_, agitation speed (rpm) and aeration (L·min^-1^) and for data storage. The pO_2 _electrode (Mettler Toledo, Switzerland) was calibrated using a pure oxygen flow as 100% value. During the fermentation process the pO_2 _inside the vessel was kept always higher than a physiological suitable value (20%) exploiting a "Gasmix" system able to enrich the air flux with pure O_2 _pulses. During the experiments the optimal pH value was kept constant at 7.5 by addition of 30% (v/v) NH_4_OH and or 30% (v/v) H_2_SO_4 _solutions through the pumps. In order to perform the microfiltration experiments two microfiltration modules were fixed vertically in the vessel, next to the baffles. The position of the modules was selected to maximize turbulence near the filtering surface thus reducing fouling occurrence [14,24]. A peristaltic pump (313U model, Watson Marlow, United Kingdom) was connected with the upper part of the module. If switched on, it produced a pressure between the inner and the outer side of the capillaries, thus driving a trans-membrane flux and the consequent removal of the exhaust medium while keeping the cells in optimal conditions inside the reactor.

### Batch experiments

Batch fermentations were performed, as previously described [8], using a shaking flask culture as inoculum. The bacterial growth was followed by measuring the absorbance at 600 nm and samples of supernatant were withdrawn during the experiments to analyse glycerol and acetate concentration in the broth. Glycerol consumption rate and acetate production and re-consumption rates were calculated.

### Microfiltration experiments

Three different types of microfiltration experiments (MF A, MF B, MF C) were performed at 37°C and at a pH of 7.5, with a starting medium volume of 1.8 L. A shaking flask culture was used as inoculum. The experiments lasted about 42-46 hours and consisted in three distinct phases: batch, fed-batch and microfiltration. After 7 hours of growth in batch mode, the fed-batch phase started by feeding the culture with a concentrated medium solution, containing 200 g·L^-1 ^of glycerol and 20 g·L^-1 ^of soya peptone, and initially using an exponential profile designed according to Keller and Dunn [25]. Successively (e.g. after 5 h of fed-batch) a linear profile was used towards a constant feeding rate calculated on the base of the consumption rate measured in previous experiments.

The feeding rates were modulated in order to keep the glycerol concentration in the broth always above 0.3 g·L^-1^. After 5 hours of fed-batch phase, at 12 hours of growth, the MF phase was started switching on the pump, connected with the modules, at a fixed set value of 30%. A total volume of exhaust medium in the range from 5.60 to 6.50 L, corresponding to about 3-folds of the initial culture volume, was exchanged during the microfiltration experiments; the permeates, coming from the microfiltration of the broth carried out by the modules, were collected. To improve the medium exchange efficiency, back-flushings of the modules were executed every hour, by simply inverting the pump flux for 1-2 minutes, using a sterilized fresh salt solution as cleaning solution. Starting from the beginning of the MF phase, a different feed, containing 420 g·L^-1 ^of glycerol and 42 g·L^-1 ^of soya peptone, was added to the medium. The three cultures were supplied with the same feeding profile up to 14 hours; starting from this time two different feeding profiles were designed and used for MF A and for MF B and C (Table 1). During the fermentation, a 100-fold diluted antifoam solution (Antifoam 204, Sigma-Aldrich, USA) was added automatically to the culture in case of high and dense foam presence. The microorganism oxygen demand was met increasing the starting stirring value from 250 to 600 rpm (MF A), to 1000 rpm (MF B) or to 800 rpm (MF C). Aeration was set in a range of values varying from a minimum of 0.55 to a maximum of 0.77 vvm (MF A), of 1.00 vvm (MF B) or of 0.88 vvm (MF C). The pO_2 _parameter was set above the 20% of dissolved oxygen for all the duration of the experiments, except for the MF C case in which the pO_2 _was kept above 40% after 15 hours of growth by increasing the airflow first and then, eventually, insufflating pure O_2 _spikes into the vessel. All the experiments were stopped after more than 40 hours of growth and only when a prolonged stationary phase occurred. The kinetics of growth were evaluated by a spectrophotometer determination of the absorbance at 600 nm of culture broth samples, withdrawn at different times during the process (Spectrophotometer DU800, Beckman Coulter, USA) and by cell dry weight values, obtained as previously described. Furthermore, during the experiments, samples of broth were collected also to evaluate the glycerol consumption, the bacterial K4 CPS and organic acids production. Samples of permeates were also withdrawn to determine the concentration of K4 polysaccharide in the microfiltrated volumes. The growth and production rates as well as the productivity were calculated for each type of experiment; the results are average values from three replicates.

### Analytical Methods

#### Sample preparation and quantification of glycerol and organic acids

Samples of 24 hour shake flask cultures or of MF fermentation broth, at different times of growth, were collected and centrifuged at 1700 × g (Avanti J-20 XP, Beckman Coulter, USA) in order to separate the biomass and recover the supernatants; samples of permeates, collected during MF experiments, were not centrifuged, not presenting cell debris. In all cases, the samples (2 mL) were then ultrafiltered/diafiltered on 10 KDa centrifugal filter devices (YM-10 Centricon, Amicon, USA) at 5000 × g and concentrated 10 folds. Filtrates were analyzed by an HPAE-PAD ionic chromatographic system (model ICS-3000, Dionex, USA) to quantify the residual glycerol and by HPLC system (model STH 575, Dionex, USA) to measure the organic acids produced, as previously reported [8]. In case of acetate inhibiting threshold tests and MF experiments, the retentate, containing the K4 CPS, was analyzed by capillary electrophoresis.

#### Capsular polysaccharide determination

The K4 CPS concentration was determined by capillary electrophoresis analysis performed on a P/ACE MDQ instrument (Beckman Coulter, USA), according to the previously described method [26]. In case of acetate inhibiting threshold shake flask experiments, the K4 CPS concentration was analysed after 24 hours of growth; while during MF fermentations the K4 polysaccharide concentration was determined in both broth supernatant and in permeate samples, withdrawn at different times. In case of MF experiments the analyses allowed to build curves of the K4 CPS concentration present in the vessel (in the culture supernatant), showing the kinetics of the capsular polysaccharide production and release. Furthermore, using the data coming from the permeates, the total concentration of K4 CPS produced was calculated as the total sum of the culture supernatant and permeate K4 CPS amounts, in terms of grams, divided by the internal, constant broth volume.

## Abbreviations

CS: Chondrotin sulphate; DO: Dissolved oxygen; FB: Fed-batch; GAGs: Glycosaminoglycans; HA: Hyaluronic acid; HS: Heparin; K4 CPS-K4: capsular polysaccharide; MF: Microfiltration.

## Competing interests

The authors declare that they have no competing interests.

## Authors' contributions

OFR, AC and MDR produced and analyzed the experimental data; DC participated in the interpretation of the results, OFR wrote the paper; CS and MDR participated in the discussion of experimental results and in the revision of manuscript's intellectual content. All authors read and approved the final manuscript.
